# Why It Is Necessary to Use the Entire Root rather than Partial Root When Doing Contralateral C7 Nerve Transfer: Cortical Plasticity Also Matters besides the Amount of Nerve Fibers

**DOI:** 10.1155/2021/8819380

**Published:** 2021-01-04

**Authors:** Jinding Guo, Xin Zhao, Jie Lao, Kaiming Gao

**Affiliations:** ^1^Department of Hand Surgery, Huashan Hospital, Fudan University, Shanghai, China; ^2^Key Laboratory of Hand Reconstruction, Ministry of Health, Shanghai, China; ^3^Shanghai Key Laboratory of Peripheral Nerve and Microsurgery, Shanghai, China

## Abstract

Previous studies suggested that the mode of donor transection is a critical factor affecting the efficacy of the contralateral C7 (CC7) nerve transfer. Nevertheless, the mechanism underlying this phenomenon remains elusive. The aim of this study was to investigate the relationship between the division modes of the CC7 nerve and cortical functional reorganization of Sprague-Dawley rats. We hypothesized that different methods of CC7 nerve transection might induce differences in cortical functional reorganization, thus resulting in differences in surgery efficacy. BDNF, TNF-*α*/IL-6, and miR-132/134 were selected as indicators of cortical functional reorganization. No significant differences in all these indicators were noted between the entire group and the entire root+posterior division group (*P* > 0.05). BDNF and miR-132/134 levels in the entire group and the entire root+posterior division group were significantly increased compared with their levels in the posterior group and the blank control group (*P* < 0.001). In all groups, BDNF, TNF-*α*/IL-6, and miR-132/134 levels in both hemispheres initially increased and subsequently decreased until week 40. In conclusion, this study provided the evidence of dynamic changes in BDNF, TNF-*α*/IL-6, and miR-132/134 in the cortex of rats after CC7 nerve transfer using different transecting modes, demonstrating that different CC7 nerve divisions might result in different surgical effects through modulation of cortical reorganization.

## 1. Introduction

Total brachial plexus injury (TBPI) is a devastating peripheral nerve injury, which can cause paralysis of the entire upper arm. Contralateral C7 (CC7) nerve transfer is an effective neurotising procedure to restore the affected limb after TBPI [1].

The CC7 nerve root is a nerve donor with ample nerve fibers (27,000–41,000) that far exceeds its recipients, such as the median nerve and musculocutaneous nerve [2]. Thus, in recent years, partial CC7 nerve transfer, which refers to dividing a selective part of CC7 as the donor, has been applied in clinical practice. The efficacy of transferring the entire CC7 nerve root to repair the median nerve is better than that of transferring the hemi-CC7 nerve root [3–6]. The mechanism underlying this phenomenon involves transferring the whole CC7 nerve root to potentially provide more nerve fibers. However, it was also reported that the entire CC7 nerve root could be transferred to repair both median nerve and bicep branch with the satisfactory results [3, 7]. After comparing the above two situations, we found that the amount of nerve fibers received by the median nerve when transferring the entire CC7 nerve root to two recipients is similar to that of transferring the hemi-CC7 nerve root to the median nerve alone. However, the surgery results were quite different. These clinical studies inferred us that the amount of nerve fibers may not be the only factor affecting the nerve recovery.

Biologically, some rat experiments also mirrored the finding. Gao et al. have reported that electrophysiological examination including maximum amplitude, latency, and muscle tetanic contraction force showed no significant difference between transferring the entire CC7 to repair the median nerve alone and transferring the entire CC7 to two recipient nerves, which were all better than partial CC7 transferring [8]. Similar results can also be obtained in terms of counts of myelinated axons in the median nerve.

Taking all the above clinical and experimental findings into consideration, we might draw the following hypothesis: the amount of nerve fibers may not be the only factor affecting the nerve recovery. In other words, harvesting the entire CC7 nerve root achieved significantly better recovery than partial harvesting for the same recipient, even if only part of the entire root was used as the donor nerve. Other mechanisms must exist besides the nerve fiber amounts underlying this phenomenon that different modes of donor transection lead to different surgery efficacies.

Recent studies have shown that CC7 nerve root transfer induces cortical functional reorganization, which has been proven to be a critical factor affecting the surgery efficacy [9–13]. Researchers compared the cerebral plasticity of intrinsic and extrinsic hand muscles both innervated by the median nerve, demonstrating that different target muscles correlated with different functional cortical reorganization [14]. Pan et al. conducted a research indicating that different recipient nerves also correlated with different functional cortical reorganization [15]. According to these studies, we hypothesized that the different modes of donor nerve transecting in addition to recipient nerves and target muscles might also influence cortical functional reorganization and eventually cause difference in surgery efficacy.

**Figure 1:**
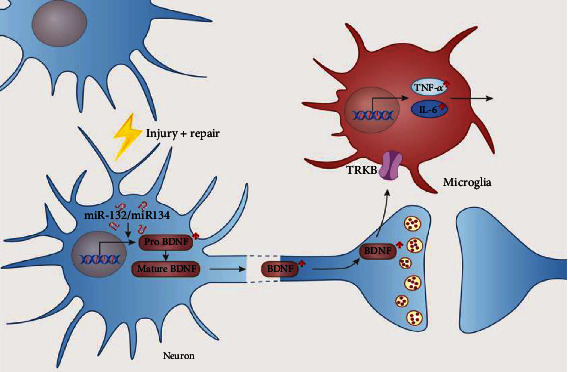
BDNF is reported to be involved in regulating cortical plasticity through activation of microglia, which secreted the proinflammatory factors such as TNF-*α* and IL-6. After injury and surgical repair of the peripheral nerve, microRNA (miR-132/134) is also reported to regulate the levels of BDNF.

During cortical functional reorganization, many neurotrophins play a critical role. Recent studies show that brain-derived neurotrophic factor (BDNF) is much abundant during all neurotrophins and plays a well-established function in modulating cortical plasticity [16, 17]. BDNF is stored in dense-core synaptic vesicles and is overexpressed and released from neurons regulating the neural plasticity. Excreted BDNF promoted the activation of astrocytes and microglia to release some inflammatory cytokines [18]. Previous studies also demonstrated that tumor necrosis factor-*α* (TNF-*α*), interleukin-6 (IL-6), and interleukin-1*β* changed dynamically with the cortical reorganization after CC7 nerve root transfer [19, 20]. In addition, levels of BDNF also are regulated by microRNA. It has been reported that miR-132 and miR-134 can regulate levels of BDNF upon the nerve injury [21]. Studies also demonstrated that miR-132 and miR-134 expression might play a fundamental role in activity-dependent synaptic plasticity [22–25]. Above all, we chose the important neurotrophin BDNF, the downstream effectors TNF-*α* and IL-6, and the upstream regulator miR-132/134 as our biomarkers (Figure 1).

In the present study, we investigated dynamic changes in BDNF, miR-132/134, and TNF-*α*/IL-6 in the motor cortex of Sprague-Dawley rats, which are critical biomarkers involved in the procedure of cortical reorganization. By comparing these critical biomarkers, we analyzed the relationship between different modes of donor nerve transection and cortical functional reorganization, thus elucidating the underlying mechanism of the different surgical outcomes.

## 2. Materials and Methods

### 2.1. Animals

Experiments were conducted on 168 adult male Sprague-Dawley (SD) rats provided by the Experiment Animal Center of Fudan University, China (license No. DF014) and weighed 200 g–250 g. The experimental procedures were performed in strict accordance with the United States National Institutes of Health Guide for the Care and Use of Laboratory Animals (NIH Publication No. 85–23, revised 1985). The Animal Ethics Committee of Fudan University of China (20150629A335) approved the study protocol. All experiments were designed to minimize the number of animals used and their suffering.

**Figure 2:**
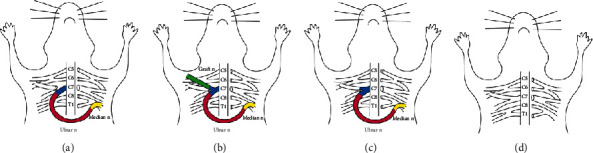
Transfer of entire root, posterior division, and entire root+posterior division. (a) In the entire root group, the entire right CC7 nerve root was transected at the trunk to the division level to be coapted with the ulnar nerve in the left side with no intension by 11-0 microsutures. (b) In the entire root+posterior division group, the entire right CC7 nerve roots were transected at the division level and only the posterior division was coapted with the ulnar nerve in the left side with no intension by 11-0 microsutures. The anterior division was transferred to the pectoralis major bridged by the medial antebrachial cutaneous nerve on the affected side. (c) In the posterior division group, only the posterior division of the right CC7 nerve root was transected and coapted with the ulnar nerve in the left side with no intension by 11-0 microsutures. (d) In the blank control group, the total left brachial plexus was avulsed and the right brachial plexus was only exposed with no operation done.

These 168 adult male SD rats all underwent root avulsion of the left brachial plexus. The rats were randomly distributed into four groups according to different division types. In the entire root group, the entire right CC7 nerve roots in 42 rats were transected to repair the left median nerve (Figure 2(a)). In the entire root+posterior division group, the entire right CC7 nerve roots in 42 rats were transected and only the posterior division was used to repair the left median nerve (Figure 2(b)). In the posterior division group, only the posterior divisions of the right CC7 nerve root in 42 rats were transected to repair the left median nerve (Figure 2(c)). In the blank control group, the total left brachial plexus was avulsed in 42 rats and the right brachial plexus was only exposed with no operation done (Figure 2(d)). In each group, we equally divided 42 rats into seven subgroups according to the time interval between the surgery and the test. These subgroups included rats tested at weeks 4 (*n* = 6), 8 (*n* = 6), 12 (*n* = 6), 16 (*n* = 6), 24 (*n* = 6), 32 (*n* = 6), and 40 (*n* = 6), respectively.

The rats were housed in plastic cages on clean sawdust, under a 12-hour light/dark cycle, with four animals per cage. The rats had free access to food and water. All rats were intraperitoneally injected preoperatively with 1% pentobarbital sodium solution (1 ml/100 g; Shanghai Reagent Company, Shanghai, China) for general anesthesia.

### 2.2. TBPI Model

Each rat was placed in the supine position. The fur of the surgery area was shaved, and the skin was prepared with iodine. According to the procedure previously reported [26], a supraclavicular transverse incision was made on the left side from the occiput to the scapular angulus superior approximately 4 cm in length to expose C_5_ to T_1_ nerve roots. Then, the nerve roots from C_5_ to T_1_ were completely avulsed from the spinal cord at the intervertebral foramen level to simulate TBPI. The avulsed nerve roots would be approximately 0.5 cm longer than before, and cerebrospinal fluid would be released to make sure the preganglionic injury. A segment approximately 2 mm in length was removed from each end of the nerves from C_5_ to T_1_. No active motion of the affected limb after surgery was considered to indicate successful establishment of the TBPI model.

### 2.3. CC7 Nerve Transfer

After TBPI, CC7 nerve transfer was performed to repair the brachial plexus. First, the proximate portion of the ulnar nerve and median nerve in the left side was explored under the axilla. The distal part of the ulnar nerve in the wrist was sectioned and moved through the subcutaneous tissue of the front chest to the contralateral body. Then, the whole CC7 nerve root was explored at the trunk to division level. Epineurotomy was performed to show the anterior and posterior division of the CC7 nerve. As per the situations in different groups, the CC7 nerve root was transected differently as noted for each group. In the entire root group, the entire root was transected and coped with the distal part of the left ulnar nerve. In the posterior division group, only the posterior division of the CC7 nerve root was transected and coped with the distal part of the left ulnar nerve. In the entire root+posterior division group, the entire root of the CC7 nerve was transected, and only the posterior division was coped with the distal part of the left ulnar nerve. The anterior division was transferred to the pectoralis major and bridged by the medial antebrachial cutaneous nerve on the left side, which could block the anterior division and avoid the anterior division growth into the ulnar nerve. Finally, in the above groups, the proximate portion of the left ulnar nerve was coped with the proximate portion of the left median nerve.

### 2.4. Obtaining Primary Motor Cortex Samples

All rats were sacrificed by decapitation under anesthesia at different time points from week 4 to week 40 after the above operations. After decapitation, intact brains were rapidly removed from the skull en bloc (all cortical layers) at ice temperature. Brain tissues between 5 mm anterior and 1 mm posterior to the Bregma and from 0.5 mm lateral to the midline to 4 mm to the midline, which are commonly recognized as the primary motor cortex (PMC), were isolated quickly for the future tests. All tissue samples were shock frozen in liquid nitrogen and stored at −80°C until use.

### 2.5. Western Blot

After the tissues of the brain were harvested for western blot, PMC areas were lysed and extracted in a radioimmunoprecipitation (RIPA) buffer containing protease (Protease Inhibitor Cocktail Set I; Calbiochem, Darmstadt, Germany) and phosphatase inhibitors according to the manufacturer's instructions at 4°C. Then, the protein concentrations were determined by the BCA standard. After the preparation of PAGE gels, we loaded the samples, markers, and the loading control according to the comparison demand. Then, protein samples were separated by SDS-PAGE in 12% polyacrylamide gels and electroblotted onto a nitrocellulose membrane (Millipore, USA). After blocking in buffer for 2 h at room temperature, membranes were incubated with rabbit anti-BDNF antibody (1 : 1000) from Abcam, UK. Then, after being washed with PBST for 3 times, membranes were incubated at room temperature for 2 h with the corresponding horseradish peroxidase-conjugated secondary antibody diluted 1 : 1000 in blocking buffer. After washing, signals were detected by enhanced chemiluminescence (ECL Plus; Amersham Biosciences) on Kodak Biomax Light films. The housekeeping gene glyceraldehyde-3-phosphate dehydrogenase (GAPDH) levels were used as the loading control. The assays were performed thrice independently.

### 2.6. Enzyme-Linked Immunosorbent Assay (ELISA)

For ELISA, rat PMC tissues were weighed and homogenized with an eightfold volume of RIPA buffer containing proteinase and phosphatase inhibitors, sonicated, and centrifuged at 14,000 g for 10 min to obtain the supernatant. Lysate protein concentrations were measured using a BCA protein assay kit (Pierce, Rockford, Illinois, USA). TNF-*α* and IL-6 levels in the rat PMC were determined using commercially available rat ELISA kits for TNF-*α* (R&D Systems, Minneapolis, Minnesota, USA) and IL-6 (R&D Systems), respectively, according to the manufacturer's instructions. The brief procedures included firstly obtaining a 96-well plate, rinsing 3 times with PBS, adding 50 *μ*l of 1 mg/ml of antigen, and incubating for 30 min at 37°C. Then, add 50 *μ*l of antibody and incubate 30 min at 37°C. After incubation, we added 50 *μ*l of goat anti-mouse IgG conjugate with alkaline phosphatase. Then, samples were incubated 30 min at 37°C and rinsed 3 times with HPLC water and 0.05% Tween 80 in 0.01 M PBS, pH 7.4. The last step was adding 50 *μ*l of color development reagent and checking enzyme and color product. The minimum detectable concentrations were less than 12.5 pg/ml for TNF-*α* and less than 62.5 pg/ml for IL-6. The assays were performed thrice independently.

### 2.7. Quantitative Polymerase Chain Reaction

Total RNA was extracted using TRIzol reagent (Life Technologies, Grand Island, NY) and precipitated in ethanol. Purity and concentration were assessed by agarose gel electrophoresis and ultraviolet (UV) spectrophotometry. After treatment with 10 U DNase I (TaKaRa, Shiga, Japan) at 37°C for 30 min, 2 mg total RNA was reverse-transcribed into cDNA at 42°C for 1 h using SuperScript reverse transcriptase (Invitrogen, CA) with random hexaprimers (TaKaRa, Shiga, Japan) according to the manufacturer's protocol. The GAPDH gene was used as an internal control. Rat miR-132/134 primers were purchased from RiboBio Co., Ltd. The primer sequences were as follows: rat BDNF, forward: 5′-TCATACTTCGGTTGCATGAAGG-3′; rat BDNF reverse: 5′-AGACCTCTCGAACCTGCCC-3′; GAPDH, forward: 5′-TGTTGCCATCAATGACCCCTT-3′; GAPDH reverse: 5′-CTCCACGACATACTCAGCA-3′. For polymerase chain reaction, 2 ml of 10-fold dilutions of the cDNA products was assayed using a TaKaRa Ex-Taq R-PCR kit (TaKaRa) with annealing for 5 min at 94°C, followed by 40 cycles of 94°C for 10 s, 55°C for 20 s, and 72°C for 30 s. The relative expression levels were calculated using the following formula: 2^-*Δ*CT^(ΔCt = Ct target gene–Ct GAPDH). The assays were performed thrice independently.

### 2.8. Immunofluorescence Analysis

Each rat was perfused through the heart with heparinized PBS (0.01 mol/l, pH 7.35) or 0.9% normal saline followed by 4% paraformaldehyde under sodium pentobarbital anesthetized to obtain tissue samples for immunofluorescence analysis. The brain tissue was excised, fixed in 4% paraformaldehyde overnight, and dehydrated in 20% and 30% sucrose at 4°C. Next, the brain was cut into 10 *μ*m thick sections and those containing the PMC area were collected. After three washes in PBS, sections were blocked with immunofluorescent blocking agent (Beyotime) for 1 h at 20–25°C and then incubated with primary rabbit polyclonal antibodies: ionized calcium-binding adaptor molecule 1 (Iba-1) overnight at 4°C and then incubated with fluorescein isothiocyanate-conjugated secondary antibodies for 2 h at room temperature in the dark. Measuring and imaging were performed by using a Leica fluorescence microscope (Leica DFC350 FX camera).

### 2.9. Statistical Analysis

Statistical calculations and data handling were performed using SPSS version 20.0 and GraphPad Prism5 (San Diego, California, USA). An analyst who was blind to animal injury performed statistical calculations and data handling. Data were expressed as the mean ± SD. The statistical significance of the data from each biomarker (BDNF, miR-132/134, TNF-*α*, and IL-6) in the motor cortex among different subgroups was determined by one-way ANOVA. Difference between the left and right sides was compared by the Student *t*-test. Least significance difference (LSD) was used for multiple comparison. The Shapiro-Wilk test was used to test data normality. Nonnormal data were analyzed using the Mann-Whitney test. Significance was set at *P* = 0.05.

## 3. Results

### 3.1. BDNF Levels in the Rat PMC after CC7 Nerve Root Transfer

**Figure 3:**
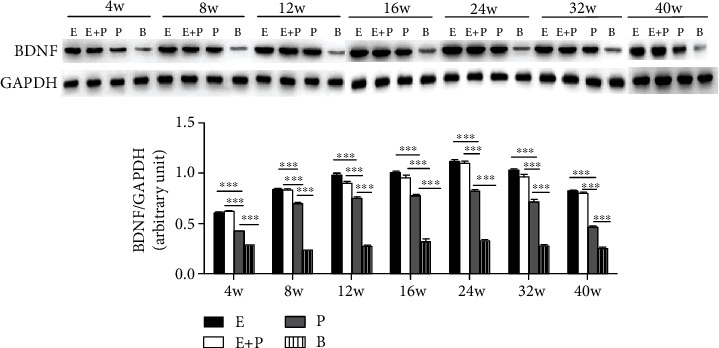
Western blot analysis of BDNF protein levels (normalized to GAPDH) in three groups in the left hemispheres. E: entire root group; E+P: entire root+posterior division group; P: posterior division group; B: blank control group. Data are expressed as mean ± SD; ^∗∗∗^*P* < 0.001 (one-way ANOVA followed by LSD).

**Figure 4:**
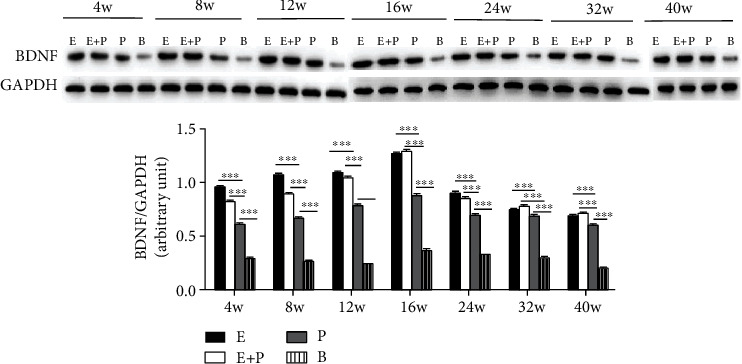
Western blot analysis of BDNF protein levels (normalized to GAPDH) in three groups in the right hemispheres. E: entire root group; E+P: entire root+posterior division group; P: posterior division group; B: blank control group. Data are expressed as mean ± SD; ^∗∗∗^*P* < 0.001 (one-way ANOVA followed by LSD).

First, western blot showed the dynamic change in BDNF levels in four groups after CC7 nerve transfer. In all groups, BDNF levels in the left hemisphere increased from week 4 to 24 and then decreased until week 40 (Figure 3). Right hemisphere results showed a similar trend. BDNF levels in the right hemisphere increased from week 4 to 16 and then decreased until week 40 (Figure 4).

Comparisons in the left PMC between different groups showed no significant difference between the entire root and entire root+posterior division groups from week 8 to 40 (*P* > 0.05). The relative amount of BDNF levels normalized to GAPDH in the entire root group was 0.67 ± 0.009, 0.84 ± 0.01, 0.97 ± 0.02, 1.00 ± 0.02, 1.11 ± 0.02, 1.03 ± 0.01, and 0.82 ± 0.004 at weeks 4, 8, 12, 16, 24, 32, and 40, respectively, and 0.62 ± 0.007, 0.83 ± 0.01, 0.90 ± 0.01, 0.96 ± 0.02, 1.10 ± 0.01, 0.96 ± 0.02, and 0.80 ± 0.003 in the entire root+posterior division group, respectively. However, BDNF levels in the above two groups were significantly increased compared with those in the posterior division group and the blank control group at all weeks analyzed after surgery (*P* < 0.001). BDNF levels in the posterior division group were 0.43 ± 0.004, 0.70 ± 0.006, 0.75 ± 0.008, 0.77 ± 0.01, 0.82 ± 0.01, 0.72 ± 0.02, and 0.46 ± 0.004, respectively, which were greater than those in the blank control group (*P* < 0.001) (Figure 3). BDNF levels in the blank control group were 0.29 ± 0.014, 0.23 ± 0.006, 0.27 ± 0.010, 0.31 ± 0.034, 0.32 ± 0.015, 0.27 ± 0.013, and 0.25 ± 0.008, respectively. We obtained similar results for the right PMC (Figure 4). No significant difference in the relative amount of BDNF levels normalized to GAPDH in the right PMC was observed between the entire root group and the entire root+posterior division group (*P* > 0.05), and these levels were significantly increased compared with those in the posterior division group (*P* < 0.001). The BDNF levels normalized to GAPDH in the right PMC in the posterior division group were also greater than those in the blank control group (*P* < 0.001).

**Figure 5:**
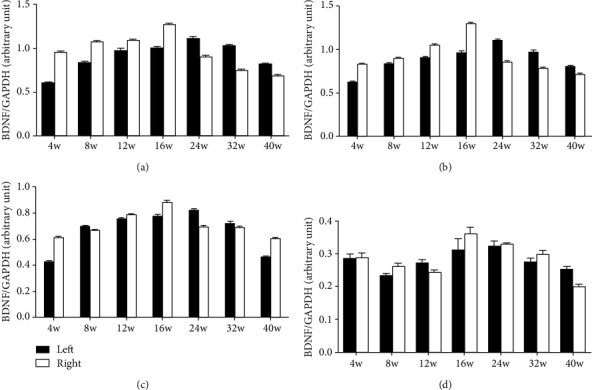
Western blot shows the comparison of the BDNF protein levels (normalized to GAPDH) between the left and right hemispheres. No significant difference was found between the left and right hemispheres. Data are expressed as mean ± SD (Student's *t*-test): (a) entire root group, (b) entire root+posterior division group, (c) posterior division group, and (d) blank control group.

Figure 5 also showed comparisons of BDNF levels between the left and right hemispheres and no significant difference of two hemispheres was noted at each time point after the operation (*P* > 0.05).

**Figure 6:**
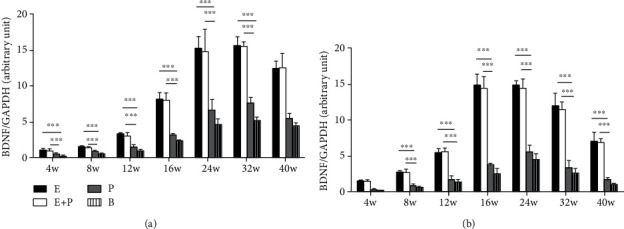
RT-PCR analysis of BDNF gene fold change levels (normalized to GAPDH) in different groups. E: entire root group; E+P: entire root+posterior division group; P: posterior division group; B: blank control group. (a) Left hemisphere; (b) right hemisphere. ^∗∗∗^*P* < 0.001. Data are expressed as mean ± SD (one-way ANOVA followed by LSD).

As shown in Figure 6(a), BDNF mRNA levels in the left PMC of rats revealed no significant difference between the entire root group and the entire root+posterior division group at all weeks analyzed after surgery (*P* > 0.05). BDNF gene expression levels increased 1.02-, 1.48-, 3.29-, 8.03-, 15.03-, 15.55-, and 12.34-fold at weeks 4, 8, 12, 16, 24, 32, and 40, respectively, in the entire root group and increased 0.93-, 1.35-, 3.02-, 7.98-, 14.68-, 15.47-, and 12.45-fold in the entire root+posterior division group. Significantly lower BDNF gene expression was observed from week 4 to week 32 in the posterior division group and the blank control group. Levels of the posterior division group increased 0.44-, 0.80-, 1.36-, 3.12-, 6.58-, and 7.53-fold, respectively (*P* < 0.001). We also obtained similar results in the right PMC of rats (Figure 6(b)).

### 3.2. MiR-132 and miR-134 Levels in the Rat PMC after CC7 Nerve Root Transfer

**Figure 7:**
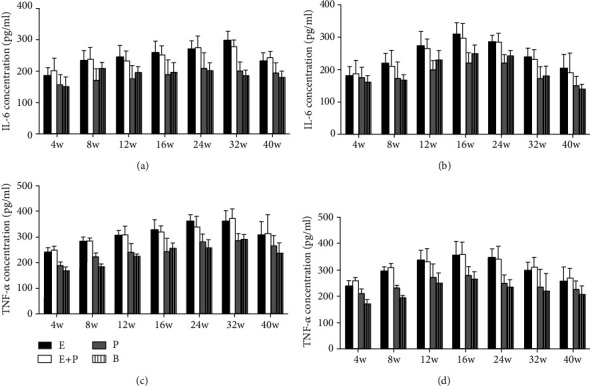
RT-PCR analysis of miR-132 and miR-134 (normalized to U6) in different groups. (a) miR-132 level of the left hemisphere. (b) miR-132 level of the right hemisphere. (c) miR-134 level of the left hemisphere. (d) miR-134 level of the right hemisphere. E: entire root group; E+P: entire root+posterior division group; P: posterior division group; B: blank control group. ^∗∗∗^*P* < 0.001. Data are expressed as mean ± SD (one-way ANOVA followed by LSD).

Comparisons of miR-132 levels between different groups revealed no significant difference between the entire root and entire root+posterior division groups (*P* > 0.05). Specifically, miR-132 levels in the right PMC in the entire root group increased 1.08-, 2.74-, 6.80-, 10.80-, 15.02-, 16.68-, and 13.95-fold at weeks 4, 8, 12, 16, 24, 32, and 40, respectively, and increased 1.17-, 2.62-, 6.97-, 11.22-, 15.50-, 16.98-, and 13.47-fold in the entire root+posterior division group. The expression levels in these two groups were greater than those in the posterior division group and the blank control group at all weeks analyzed after surgery. The relative levels of miR-132 were 0.33-, 1.29-, 2.42-, 4.59-, 5.82-, 6.25-, and 5.39-fold, respectively, in the posterior division group and 0.21-, 0.93-, 1.70-, 3.02-, 3.67-, 4.98-, and 4.33-fold change in the blank control group (Figure 7(a)). However, significant differences were only noted at weeks 12, 16, 24, 32, and 40 (*P* < 0.001). RT-PCR of miR-132 also showed similar results in the left PMC (Figure 7(b)).

In addition, miR-134 levels were compared between the entire root group and the entire root+posterior division group, and no significant difference between them was observed (*P* > 0.05). Specifically, miR-134 levels in the right PMC in the entire root group increased 1.02-, 4.26-, 6.66-, 8.18-, 12.42-, 13.71-, and 10.65-fold at weeks 4, 8, 12, 16, 24, 32, and 40, respectively, and increased 1.11-, 4.22-, 6.74-, 8.43-, 11.77-, 13.37-, and 10.16-fold in the entire root+posterior division group, respectively. Reduced miR-134 expression was observed in the posterior division group and the blank control group (Figure 7(c)). The relative levels were 0.57-, 1.96-, 3.41-, 4.69-, 5.94-, 6.91-, and 5.14-fold in the posterior division group and 0.33-, 1.30-, 2.11-, 2.98-, 3.78-, 4.98-, and 4.26-fold in the blank control group, respectively. However, significant differences were only noted at weeks 8 and 16 (*P* < 0.001). Similar results were also observed in the left PMC (Figure 7(d)).

### 3.3. TNF-*α* and IL-6 Levels in the Rat PMC after CC7 Nerve Root Transfer

**Figure 8:**
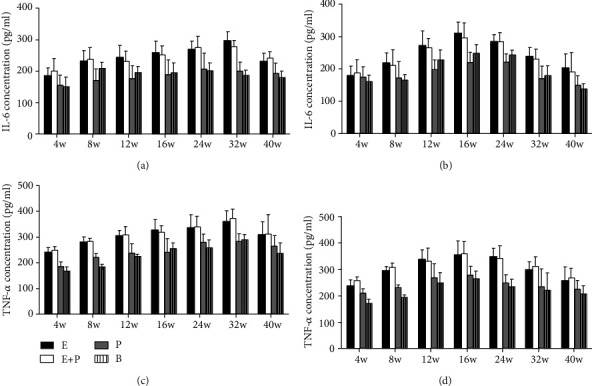
ELISA analysis of IL-6 and TNF-*α* concentration in different groups. (a) IL-6 concentration of the left hemisphere. (b) IL-6 concentration of the right hemisphere. (c) TNF-*α* concentration of the left hemisphere. (d) TNF-*α* concentration of the right hemisphere. Samples were obtained from rats subjected to different divisions of CC7 nerve root at 4 w, 8 w, 12 w, 16 w, 24 w, 32 w, and 40 w postsurgery. E: entire root group; E+P: entire root+posterior division group; P: posterior division group; B: blank control group. Data are expressed as mean ± SD (one-way ANOVA followed by LSD).

No significant difference of IL-6 levels was observed between the entire root group and the entire root+posterior division group in the left PMC (*P* > 0.05). IL-6 levels were 184.5 ± 25.1, 231.7 ± 32.7, 243.5 ± 36.9, 257.2 ± 36.8, 268.0 ± 26.6, 296.2 ± 28.6, and 230.1 ± 26.5 in the entire root group and 200.8 ± 39.0, 236.4 ± 37.2, 230.5 ± 32.2, 251.4 ± 27.7, 274.6 ± 35.5, 277.0 ± 20.9, and 241.0 ± 20.4 in the entire root+posterior division group at weeks 4, 8, 12, 16, 24, 32, and 40, respectively (unit: pg/ml). IL-6 levels in the two groups were increased compared with those in the posterior division group and the blank control group. The levels in the posterior group were 165.0 ± 31.5, 219.0 ± 36.8, 206.5 ± 41.8, 207.5 ± 46.9, 216.5 ± 50.4, 199.4 ± 28.3, and 192.7 ± 31.6, respectively (unit: pg/ml) (Figure 8(a)). However, the difference was not significant (*P* > 0.05). Similar results in the right PMC were observed (Figure 8(b)).

No significant difference in TNF-*α* levels was observed between the entire root group and the entire root+posterior division group in the left PMC (*P* > 0.05). TNF-*α* levels were 249.5 ± 17.9, 291.2 ± 18.2, 302.7 ± 22.0, 326.4 ± 40.1, 347.8 ± 47.9, 369.7 ± 41.6, and 307.4 ± 51.8 in the entire root group and 258.4 ± 14.2, 274.3 ± 10.4, 306.2 ± 33.5, 319.5 ± 23.1, 328.1 ± 41.4, 390.3 ± 37.7, and 322.4 ± 73.1 in the entire root+posterior division group at weeks 4, 8, 12, 16, 24, 32, and 40, respectively (unit: pg/ml). TNF-*α* levels in the two groups were increased compared with those in the posterior division group and the blank control group. The levels of the posterior division group were 205.8 ± 16.3, 251.8 ± 13.2, 267.7 ± 35.3, 280.7 ± 52.6, 299.5 ± 30.8, 323.6 ± 28.9, and 274.6 ± 40.9 (unit: pg/ml) (Figure 8(c)). However, the difference was also not significant (*P* > 0.05). Similar results in the right PMC were obtained (Figure 8(d)).

### 3.4. Microglia Cell Counting

**Figure 9:**
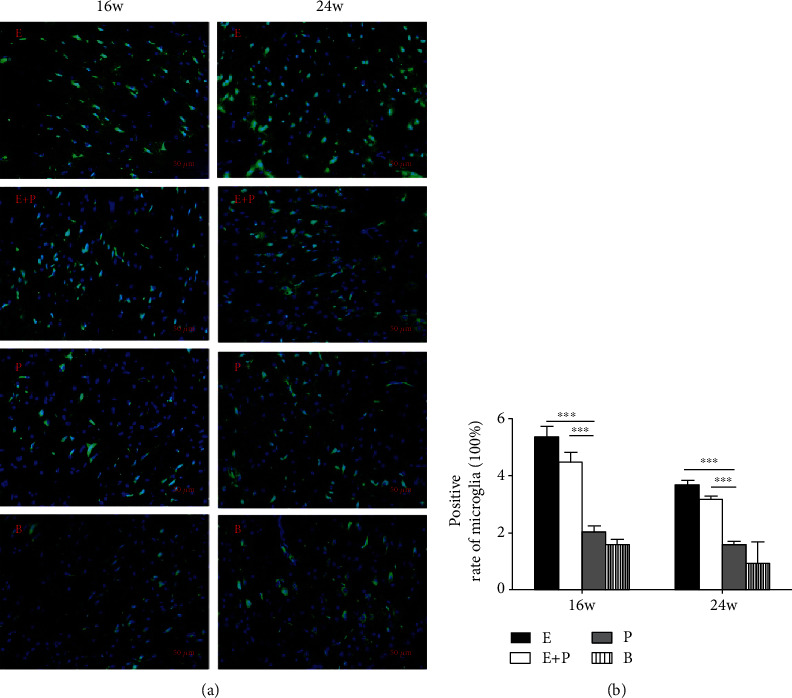
Immunofluorescence staining assay of microglia in the right PMC in 16 w and 24 w after surgery in different groups. (a) Immunofluorescence staining of Iba-1 (green). Scale bar = 50 *μ*m. (b) Positive rate of microglia cells in different groups. E: Iba-1 expression of the entire root group; E+P: Iba-1 expression of the entire root+posterior division group; P: Iba-1 expression of the posterior division group; B: Iba-1 expression of the blank control group. ^∗∗∗^*P* < 0.001. Data are expressed as mean ± SD (one-way ANOVA followed by LSD).

Although no significant difference was found in terms of proinflammatory factors, we have also investigated the microglia cells in the PMC in different groups through the immunofluorescence analysis. Considering that the contralateral brain cortical eventually took control of the injured arm in rats, we chose the right PMC in 16 w and 24 w after surgery to compare the microglia cells in different groups. Images showed that there was no significant difference in terms of the positive rates of microglia cells between the entire root group and the entire root+posterior division group, which were greater than the posterior division group and the blank control group (*P* < 0.001) (Figure 9).

## 4. Discussion

Peripheral nerve injury is also related to the central nervous system, especially for the TBPI. On the one hand, some patients are accompanied with the central nervous system injury. On the other hand, the signals received from the peripheral system are altered after the TBPI. Functional cortical reorganization occurring in the central nervous system is considered an important factor influencing the surgery efficacy [9, 17, 20, 27, 28]. The present study provides insight into the possible relationship between different division modes of CC7 nerve root and cortical reorganization by comparing the dynamic levels of these molecules, such as neurotrophins, miR-RNA, and the inflammatory cytokines. To our knowledge, this is the first study exploring the underlying mechanism of this phenomenon.

BDNF is highly expressed in the central nervous system and involved in numerous neuroplasticity processes, such as neuronal survival and neurogenesis [16, 29, 30]. In our experiments, we first analyzed the dynamic levels of BDNF after CC7 nerve transfer. In all groups and hemispheres, BDNF levels exhibited an increasing trend that peaked at weeks 16 to 24 and decreased until week 40. This finding was consistent with the cortical reorganization and demonstrated that BDNF represents an indicator of functional cortical reorganization. During the early phase after the surgery, the peripheral neural route was built, and the signals received from the peripheral stimuli were gradually strengthened. BDNF levels increased with the changes in neuroplasticity and functional cortical reorganization. During the late phase, functional cortical reorganization was almost complete, and some negative regulators start to inhibit BDNF overexpression [17, 31]. Thus, BDNF levels decreased during the late phase.

BDNF levels were also compared between different CC7 division modes. No significant differences were noted between the entire root group and the entire root+posterior division group. Comparing the two groups, the way of CC7 nerve transection was identical, but nerve fiber amount used as the donor nerve differed. According to previous studies, BDNF is an important biomarker of cortical reorganization [16, 17, 32, 33]. We could infer that the same CC7 transection method yielded the same level of cortical reorganization, and no significant differences in the surgical efficacy were noted. However, BDNF levels of them were all greater than those of the posterior division group. When we compared the entire root+posterior division group and the posterior division group, we found that the number of donor nerve fibers in both groups was similar, but the mode of CC7 transection differed. In the posterior division group, only the posterior division was transected. In the entire root+posterior division group, the entire nerve root was transected. Given the role of BDNF in cortical reorganization, we could infer that different methods of CC7 transection yielded different levels of cortical reorganization and eventually changed the surgical efficacy. We also found that BDNF levels of the posterior division group were greater than those in the blank control group (TBPI), which meant that BDNF levels were upregulated after the surgery to repair the injury and the cortical reorganization did happen after the CC7 nerve transfer.

Then, we compared BDNF levels in both hemispheres at every time point after surgery. No significant difference was noted between both hemispheres. However, we observed that the peak times of the right and left hemispheres were not the same. The peak time point of the right hemisphere was week 16, which was earlier than that noted for the left one at week 24. It is possible that after surgery, the right hemisphere first accepted the peripheral signals for the nerve injury and then the left hemisphere gradually took charge of the affected limb for the rerouted neural pathway [9, 27, 34].

In addition to BDNF, a large body of evidence demonstrates that proinflammatory cytokines play an important role in transhemispheric functional reorganization [19] [20]. In our experiments, these two proinflammatory cytokines were compared between different groups. No significant difference was noted between the entire root group and the entire root+posterior division group, and the values in these groups were increased compared with those in the posterior division and blank control groups. TNF-*α* and IL-6 results were similar to those noted for BDNF, and these results also revealed the relationship between the type of CC7 division and functional cortical reorganization. Besides, the dynamic change of TNF-*α* and IL-6 may also be accounted for the underlying mechanism that BDNF was involved in transhemispheric functional reorganization. Upon nerve injury, inflammatory cells are activated in the neural network and synaptic junctions in the central nervous system. In our experiment, immunofluorescence analysis showed that the microglia cells were activated in the entire root group and the entire root+posterior division group in 16 w and 24 w. The activated inflammatory cells secrete inflammatory cytokines or mediators that act on neurons and regulate the development of cortical reorganization [35–37]. When more CC7 nerve was harvested, more corresponding representations in the motor cortex were replaced and more inflammatory cytokines were secreted [38, 39]. TNF-*α* and IL-6 in turn play potential roles in the neural circuitry and synaptic plasticity, which may facilitate the cortical reorganization and functional recovery of the affected limb [40, 41]. Although the statistical analysis indicated that the difference was not significant, we believed more research about the relationship between proinflammatory cytokines and the cortical reorganization after CC7 nerve root transfer should be performed.

miR-132 and miR-134 are brain-enriched miRNAs with a well-documented function in neuronal plasticity [22, 42–44]. Dynamic miR-132 and miR-134 levels were similar to BDNF, providing additional evidences indicating that differences in cortical reorganization after different CC7 nerve root divisions modulated surgery efficacy. Studies have shown that miR-132 expression is activity dependent, which may contribute to the long-lasting proteomic changes required for experience-dependent neuronal plasticity [45]. Our results further supported the association between BDNF and miR-134. In our study, we reported that BDNF levels were related with the division mode of the CC7 nerve transfer. However, the underlying mechanism whereby CC7 nerve division regulates BDNF levels remains unknown. Previous studies reported that miR-132 and miR-134 are involved in BDNF regulation [39, 46, 47]. They may also be associated with BDNF expression after the CC7 nerve root transfer. However, the hypothesis must be proven based on additional experiments.

Moreover, we have noticed that biomarkers of the right motor cortex were not fairly consistent among groups although the left avulsion injuries were identical among groups. The results were in accordance with the process of transcortical reorganization which includes both hemispheres. The contralateral cortex firstly lost the control of the injured side and then the ipsilateral cortex took over the control after the connection of CC7 nerve transfer. Then, after the process of reorganization, the contralateral cortex finally regained the control of the injured arm.

Many factors influenced the final efficacy of CC7 transfer including the target muscle, transfer route, donor nerve, and functional cortical reorganization. Functional cortical reorganization occurring after CC7 nerve transfer is a much complex biological process involving many regulators. In the current study, we have found the evidence that division mode could affect the surgery efficacy not only by donor nerve fibers but also by the functional cortical reorganization and these correlated biomarkers could be the targets of future investigation to fully utilize the potential efficacy of the CC7 nerve transfer.

## 5. Conclusion

In conclusion, the present study compared the different levels of BDNF, TNF-*α*, IL-6, and miR-132/134 in rat PMC after CC7 nerve root transfer in different division groups. Results demonstrated that different divisions of CC7 nerve may result in different surgical effects through the modulation of the cortical reorganization. Additional studies are required to further elucidate the further role of these biomarkers in cortical reorganization and their surgical effects.

## Data Availability

The underlying data supporting the results of our study could be obtained by contacting the corresponding author whose email has been provided in the manuscript.
